# 2-{[(2-Hy­droxy-3,5-dimethyl­benz­yl)(meth­yl)amino]­meth­yl}-4,6-dimethyl­phenol

**DOI:** 10.1107/S1600536812022180

**Published:** 2012-05-19

**Authors:** Chatchai Veranitisagul, Worawat Wattanathana, Attaphon Kaewvilai, Tanwawan Duangthongyou, Apirat Laobuthee, Nattamon Koonsaeng

**Affiliations:** a Department of Materials and Metallurgical Engineering, Faculty of Engineering, Rajamangala University of Technology Thanyaburi, Pathumthani 12110, Thailand; b Department of Chemistry, Faculty of Science, Kasetsart University, Bangkok 10900, Thailand; c Department of Materials Engineering, Faculty of Engineering, Kasetsart University, Bangkok 10900, Thailand

## Abstract

In the title compound, C_19_H_25_NO_2_, the dihedral angle between the benzene rings is 53.15 (8)°. One of the –OH groups forms an intra­molecular O—H⋯N link, generating an *S*(6) ring. The other –OH group forms an inter­molecular O—H⋯N hydrogen bond in the crystal, generating centrosymmetric *R*_2_^2^(20) loops.

## Related literature

For the synthesis, see: Chirachanchai *et al.* (2009 ▶). For metal-responsive properties of *N*,*N*-bis­(2-hy­droxy­benz­yl)alkyl­amines, see: Veranitisagul *et al.* (2011 ▶). For the use of *N*,*N*-bis­(2-hy­droxy­benz­yl)alkyl­amines in the synthesis of macrocyclic mol­ecules, see: Rungsimanon *et al.* (2008 ▶).
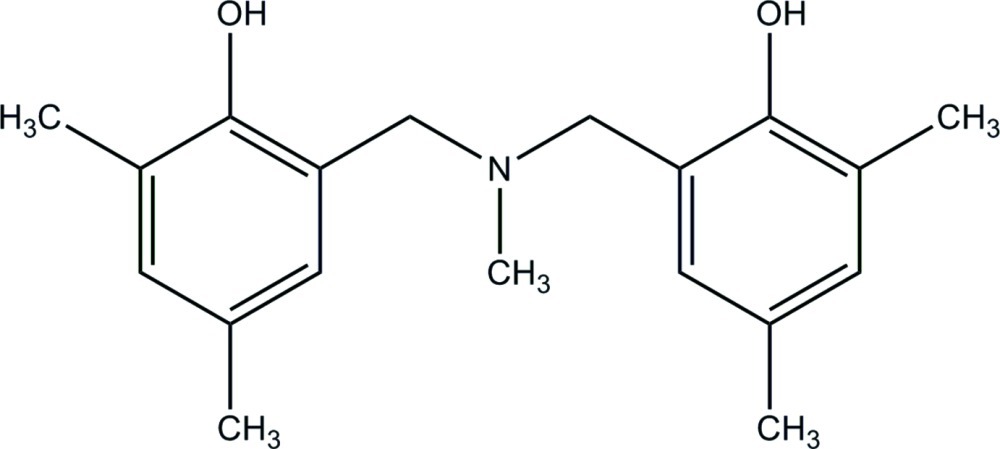


## Experimental

### 

#### Crystal data

C_19_H_25_NO_2_
*M* *_r_* = 299.40
Triclinic, 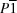

*a* = 5.4598 (4) Å
*b* = 12.3285 (12) Å
*c* = 13.0441 (13) Å
α = 95.412 (3)°
β = 96.482 (2)°
γ = 94.238 (2)°
*V* = 865.33 (14) Å^3^
*Z* = 2
Mo *K*α radiation
μ = 0.07 mm^−1^
*T* = 296 K
0.52 × 0.30 × 0.24 mm

#### Data collection

Siemens P4 diffractometer
6613 measured reflections
3944 independent reflections
2722 reflections with *I* > 2σ(*I*)
*R*_int_ = 0.021

#### Refinement

*R*[*F* ^2^ > 2σ(*F* ^2^)] = 0.050
*wR*(*F* ^2^) = 0.182
*S* = 1.06
3944 reflections
299 parameters
All H-atom parameters refined
Δρ_max_ = 0.27 e Å^−3^
Δρ_min_ = −0.26 e Å^−3^

### 

Data collection: *XSCANS* (Siemens, 1992 ▶); cell refinement: *XSCANS*; data reduction: *XSCANS*; program(s) used to solve structure: *SHELXS97* (Sheldrick, 2008 ▶); program(s) used to refine structure: *SHELXL97* (Sheldrick, 2008 ▶); molecular graphics: *SHELXTL* (Sheldrick, 2008 ▶); software used to prepare material for publication: *SHELXL97*.

## Supplementary Material

Crystal structure: contains datablock(s) I, global. DOI: 10.1107/S1600536812022180/hb6793sup1.cif

Structure factors: contains datablock(s) I. DOI: 10.1107/S1600536812022180/hb6793Isup2.hkl

Supplementary material file. DOI: 10.1107/S1600536812022180/hb6793Isup3.cml

Additional supplementary materials:  crystallographic information; 3D view; checkCIF report

## Figures and Tables

**Table 1:** Hydrogen-bond geometry (Å, °)

*D*—H⋯*A*	*D*—H	H⋯*A*	*D*⋯*A*	*D*—H⋯*A*
O1—H′⋯N	1.03 (3)	1.71 (3)	2.6895 (18)	156 (2)
O2—H′′⋯O1^i^	0.94 (3)	2.02 (4)	2.9114 (18)	158 (2)

Symmetry code: (i) 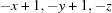.

**Fig. 1.**
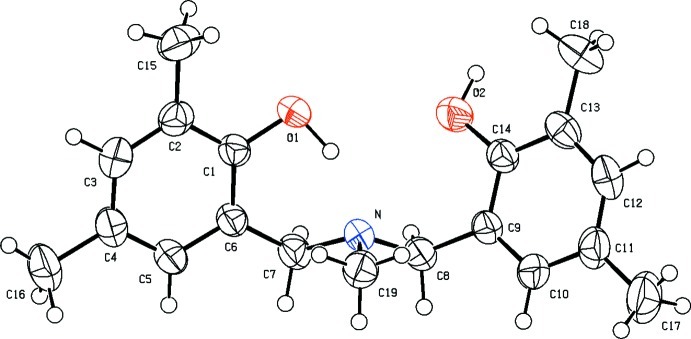
Molecular structure of the title compound (arbitrary spheres for the H atoms).

**Fig. 2.**
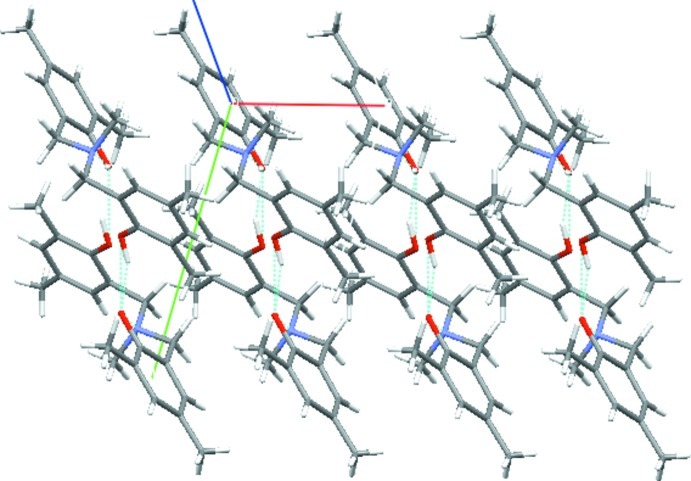
The packing structure of compound (Hydrogen bonds indicated by dash lines).

## supplementary crystallographic information

### Experimental

The preparation of the title compound was reported elsewhere (Chirachanchai *et al.*, 2009). Colorless blocks were recrystallized from 2-propanol solution.

### Refinement

All H atoms were located in difference maps and freely refined.

### Crystal data

**Table:**

C_19_H_25_NO_2_	*Z* = 2
*M**_r_* = 299.40	*F*(000) = 324
Triclinic, *P*1	*D*_x_ = 1.149 Mg m^−^^3^
*a* = 5.4598 (4) Å	Mo *K*α radiation, λ = 0.71073 Å
*b* = 12.3285 (12) Å	Cell parameters from 2370 reflections
*c* = 13.0441 (13) Å	θ = 3.2–27.2°
α = 95.412 (3)°	µ = 0.07 mm^−^^1^
β = 96.482 (2)°	*T* = 296 K
γ = 94.238 (2)°	Block, colorless
*V* = 865.33 (14) Å^3^	0.52 × 0.30 × 0.24 mm

### Data collection

**Table:**

Siemens P4 diffractometer	2722 reflections with *I* > 2σ(*I*)
Radiation source: fine-focus sealed tube	*R*_int_ = 0.021
Graphite monochromator	θ_max_ = 27.6°, θ_min_ = 1.6°
ω scans	*h* = −6→7
6613 measured reflections	*k* = −16→15
3944 independent reflections	*l* = −16→16

### Refinement

**Table:**

Refinement on *F*^2^	Primary atom site location: structure-invariant direct methods
Least-squares matrix: full	Secondary atom site location: difference Fourier map
*R*[*F*^2^ > 2σ(*F*^2^)] = 0.050	Hydrogen site location: inferred from neighbouring sites
*wR*(*F*^2^) = 0.182	All H-atom parameters refined
*S* = 1.06	*w* = 1/[σ^2^(*F*_o_^2^) + (0.1149*P*)^2^] where *P* = (*F*_o_^2^ + 2*F*_c_^2^)/3
3944 reflections	(Δ/σ)_max_ < 0.001
299 parameters	Δρ_max_ = 0.27 e Å^−^^3^
0 restraints	Δρ_min_ = −0.26 e Å^−^^3^

### Special details

**Table:**

Geometry. All e.s.d.'s (except the e.s.d. in the dihedral angle between two l.s. planes) are estimated using the full covariance matrix. The cell e.s.d.'s are taken into account individually in the estimation of e.s.d.'s in distances, angles and torsion angles; correlations between e.s.d.'s in cell parameters are only used when they are defined by crystal symmetry. An approximate (isotropic) treatment of cell e.s.d.'s is used for estimating e.s.d.'s involving l.s. planes.
Refinement. Refinement of *F*^2^ against ALL reflections. The weighted *R*-factor *wR* and goodness of fit *S* are based on *F*^2^, conventional *R*-factors *R* are based on *F*, with *F* set to zero for negative *F*^2^. The threshold expression of *F*^2^ > σ(*F*^2^) is used only for calculating *R*-factors(gt) *etc*. and is not relevant to the choice of reflections for refinement. *R*-factors based on *F*^2^ are statistically about twice as large as those based on *F*, and *R*- factors based on ALL data will be even larger.

### Fractional atomic coordinates and isotropic or equivalent isotropic displacement parameters (Å^2^)

**Table:**

	*x*	*y*	*z*	*U*_iso_*/*U*_eq_	
O1	0.5039 (2)	0.38818 (10)	0.16018 (10)	0.0517 (3)	
N	0.2371 (2)	0.21998 (11)	0.04228 (10)	0.0404 (3)	
C1	0.4085 (3)	0.34523 (12)	0.24228 (13)	0.0427 (4)	
C14	0.4502 (3)	0.30149 (13)	−0.15535 (12)	0.0428 (4)	
C10	0.3116 (4)	0.11247 (15)	−0.20468 (13)	0.0532 (4)	
C2	0.5258 (3)	0.37697 (14)	0.34231 (14)	0.0479 (4)	
C6	0.1937 (3)	0.27292 (13)	0.22555 (12)	0.0423 (4)	
C9	0.2935 (3)	0.20900 (14)	−0.14369 (12)	0.0435 (4)	
C3	0.4254 (3)	0.33434 (15)	0.42452 (14)	0.0529 (4)	
C7	0.0629 (3)	0.24498 (16)	0.11772 (13)	0.0487 (4)	
C5	0.1029 (3)	0.23192 (15)	0.31059 (14)	0.0496 (4)	
C13	0.6237 (3)	0.29546 (15)	−0.22569 (14)	0.0490 (4)	
C8	0.1141 (3)	0.21588 (16)	−0.06464 (13)	0.0472 (4)	
C11	0.4815 (4)	0.10371 (16)	−0.27587 (14)	0.0571 (5)	
C4	0.2141 (3)	0.26180 (15)	0.41101 (14)	0.0529 (4)	
C12	0.6338 (4)	0.19581 (17)	−0.28429 (14)	0.0556 (5)	
C19	0.3486 (4)	0.11876 (15)	0.06030 (16)	0.0532 (4)	
C16	0.1053 (6)	0.2193 (3)	0.50252 (19)	0.0796 (7)	
C15	0.7506 (4)	0.45821 (19)	0.3588 (2)	0.0640 (5)	
C18	0.7977 (4)	0.3916 (2)	−0.2386 (2)	0.0651 (6)	
C17	0.4954 (9)	−0.0025 (2)	−0.3427 (2)	0.0933 (9)	
O2	0.4257 (2)	0.39465 (10)	−0.09216 (10)	0.0585 (4)	
H5	−0.048 (3)	0.1827 (16)	0.2992 (14)	0.052 (5)*	
H12	0.751 (4)	0.1965 (19)	−0.3286 (19)	0.079 (7)*	
H7B	−0.068 (4)	0.1831 (17)	0.1203 (15)	0.061 (5)*	
H10	0.205 (4)	0.0478 (19)	−0.1977 (17)	0.067 (6)*	
H3	0.510 (4)	0.353 (2)	0.497 (2)	0.083 (7)*	
H19C	0.222 (4)	0.0535 (19)	0.0524 (17)	0.073 (6)*	
H8B	−0.017 (4)	0.1503 (18)	−0.0811 (16)	0.066 (6)*	
H8A	0.020 (3)	0.2867 (16)	−0.0664 (15)	0.056 (5)*	
H19B	0.430 (4)	0.1259 (18)	0.1319 (19)	0.069 (6)*	
H16A	−0.068 (8)	0.216 (4)	0.490 (3)	0.167 (16)*	
H19A	0.466 (4)	0.1081 (18)	0.0130 (17)	0.069 (6)*	
H18B	0.855 (4)	0.4335 (17)	−0.1729 (18)	0.059 (6)*	
H7A	−0.030 (4)	0.3078 (17)	0.0938 (15)	0.060 (5)*	
H17A	0.644 (8)	−0.001 (4)	−0.363 (4)	0.155 (17)*	
H18A	0.944 (5)	0.369 (2)	−0.257 (2)	0.091 (8)*	
H15B	0.827 (5)	0.459 (2)	0.433 (3)	0.110 (9)*	
H15C	0.718 (4)	0.535 (2)	0.335 (2)	0.088 (7)*	
H'	0.426 (5)	0.331 (2)	0.100 (2)	0.097 (8)*	
H16C	0.188 (6)	0.241 (3)	0.565 (3)	0.142 (13)*	
H18C	0.729 (4)	0.443 (2)	−0.276 (2)	0.086 (7)*	
H''	0.483 (5)	0.457 (3)	−0.122 (2)	0.104 (9)*	
H16B	0.079 (7)	0.133 (4)	0.490 (3)	0.139 (12)*	
H17C	0.424 (9)	0.000 (4)	−0.409 (5)	0.184 (18)*	
H15A	0.879 (5)	0.432 (2)	0.327 (2)	0.086 (8)*	
H17B	0.490 (6)	−0.064 (3)	−0.304 (3)	0.136 (12)*	

### Atomic displacement parameters (Å^2^)

**Table:**

	*U*^11^	*U*^22^	*U*^33^	*U*^12^	*U*^13^	*U*^23^
O1	0.0599 (7)	0.0452 (7)	0.0506 (7)	−0.0098 (5)	0.0196 (6)	0.0038 (5)
N	0.0436 (7)	0.0421 (7)	0.0366 (7)	0.0005 (5)	0.0092 (5)	0.0067 (5)
C1	0.0477 (8)	0.0374 (8)	0.0446 (9)	0.0025 (6)	0.0148 (7)	0.0026 (6)
C14	0.0455 (8)	0.0447 (9)	0.0392 (8)	0.0025 (6)	0.0059 (6)	0.0098 (7)
C10	0.0697 (11)	0.0510 (10)	0.0369 (9)	−0.0053 (9)	0.0038 (8)	0.0053 (7)
C2	0.0483 (8)	0.0428 (9)	0.0521 (10)	0.0064 (7)	0.0093 (7)	−0.0029 (7)
C6	0.0434 (8)	0.0437 (9)	0.0415 (9)	0.0027 (6)	0.0124 (6)	0.0056 (7)
C9	0.0477 (8)	0.0474 (9)	0.0355 (8)	−0.0013 (7)	0.0050 (6)	0.0089 (6)
C3	0.0585 (10)	0.0568 (11)	0.0435 (10)	0.0127 (8)	0.0054 (8)	0.0006 (8)
C7	0.0412 (8)	0.0608 (11)	0.0445 (9)	−0.0020 (8)	0.0112 (7)	0.0062 (8)
C5	0.0514 (9)	0.0518 (10)	0.0479 (10)	−0.0001 (7)	0.0159 (8)	0.0083 (8)
C13	0.0454 (8)	0.0567 (10)	0.0489 (9)	0.0067 (7)	0.0072 (7)	0.0216 (8)
C8	0.0453 (8)	0.0563 (10)	0.0392 (9)	−0.0056 (8)	0.0053 (7)	0.0081 (7)
C11	0.0812 (12)	0.0549 (11)	0.0375 (9)	0.0118 (9)	0.0101 (8)	0.0082 (8)
C4	0.0618 (10)	0.0563 (10)	0.0444 (10)	0.0097 (8)	0.0168 (8)	0.0096 (8)
C12	0.0653 (11)	0.0656 (12)	0.0429 (10)	0.0187 (9)	0.0186 (8)	0.0174 (8)
C19	0.0667 (11)	0.0461 (10)	0.0479 (10)	0.0079 (8)	0.0072 (9)	0.0075 (8)
C16	0.0953 (18)	0.100 (2)	0.0474 (12)	−0.0027 (15)	0.0207 (12)	0.0215 (12)
C15	0.0553 (11)	0.0583 (13)	0.0727 (14)	−0.0043 (9)	0.0034 (10)	−0.0104 (10)
C18	0.0531 (11)	0.0650 (13)	0.0848 (17)	0.0039 (10)	0.0231 (11)	0.0303 (13)
C17	0.160 (3)	0.0665 (16)	0.0577 (15)	0.0170 (17)	0.0367 (17)	−0.0026 (12)
O2	0.0753 (8)	0.0424 (7)	0.0598 (8)	−0.0029 (6)	0.0221 (6)	0.0052 (6)

### Geometric parameters (Å, º)

**Table:**

O1—C1	1.3750 (19)	C13—C12	1.393 (3)
O1—H'	1.04 (3)	C13—C18	1.498 (3)
N—C19	1.456 (2)	C8—H8B	1.03 (2)
N—C7	1.472 (2)	C8—H8A	1.05 (2)
N—C8	1.473 (2)	C11—C12	1.378 (3)
C1—C2	1.394 (2)	C11—C17	1.515 (3)
C1—C6	1.403 (2)	C4—C16	1.511 (3)
C14—O2	1.373 (2)	C12—H12	0.91 (2)
C14—C13	1.392 (2)	C19—H19C	1.01 (2)
C14—C9	1.405 (2)	C19—H19B	0.98 (2)
C10—C9	1.384 (2)	C19—H19A	0.94 (2)
C10—C11	1.387 (3)	C16—H16A	0.94 (4)
C10—H10	0.97 (2)	C16—H16C	0.89 (4)
C2—C3	1.387 (3)	C16—H16B	1.05 (4)
C2—C15	1.508 (3)	C15—H15B	1.01 (3)
C6—C5	1.387 (2)	C15—H15C	1.05 (3)
C6—C7	1.501 (2)	C15—H15A	0.92 (3)
C9—C8	1.501 (2)	C18—H18B	0.96 (2)
C3—C4	1.391 (3)	C18—H18A	0.92 (3)
C3—H3	1.00 (3)	C18—H18C	0.91 (3)
C7—H7B	1.01 (2)	C17—H17A	0.88 (4)
C7—H7A	1.01 (2)	C17—H17C	0.91 (6)
C5—C4	1.384 (3)	C17—H17B	0.96 (4)
C5—H5	0.973 (18)	O2—H''	0.95 (3)
			
C1—O1—H'	100.4 (15)	H8B—C8—H8A	107.3 (15)
C19—N—C7	111.00 (14)	C12—C11—C10	117.31 (17)
C19—N—C8	111.41 (14)	C12—C11—C17	121.5 (2)
C7—N—C8	110.75 (12)	C10—C11—C17	121.2 (2)
O1—C1—C2	118.74 (14)	C5—C4—C3	117.52 (16)
O1—C1—C6	120.42 (15)	C5—C4—C16	121.06 (19)
C2—C1—C6	120.82 (14)	C3—C4—C16	121.40 (19)
O2—C14—C13	123.18 (14)	C11—C12—C13	123.26 (16)
O2—C14—C9	116.30 (14)	C11—C12—H12	122.7 (15)
C13—C14—C9	120.50 (16)	C13—C12—H12	114.1 (15)
C9—C10—C11	122.18 (17)	N—C19—H19C	112.4 (12)
C9—C10—H10	120.0 (13)	N—C19—H19B	108.3 (13)
C11—C10—H10	117.8 (13)	H19C—C19—H19B	107.1 (18)
C3—C2—C1	118.13 (15)	N—C19—H19A	108.2 (13)
C3—C2—C15	121.91 (17)	H19C—C19—H19A	110.3 (18)
C1—C2—C15	119.93 (17)	H19B—C19—H19A	110.5 (18)
C5—C6—C1	118.57 (15)	C4—C16—H16A	109 (3)
C5—C6—C7	121.62 (14)	C4—C16—H16C	116 (2)
C1—C6—C7	119.77 (14)	H16A—C16—H16C	123 (3)
C10—C9—C14	118.83 (15)	C4—C16—H16B	109 (2)
C10—C9—C8	121.39 (15)	H16A—C16—H16B	84 (3)
C14—C9—C8	119.77 (15)	H16C—C16—H16B	111 (3)
C2—C3—C4	122.74 (17)	C2—C15—H15B	107.5 (17)
C2—C3—H3	119.9 (13)	C2—C15—H15C	114.4 (13)
C4—C3—H3	117.3 (13)	H15B—C15—H15C	116 (2)
N—C7—C6	111.73 (13)	C2—C15—H15A	111.8 (16)
N—C7—H7B	113.6 (11)	H15B—C15—H15A	98 (2)
C6—C7—H7B	107.5 (12)	H15C—C15—H15A	109 (2)
N—C7—H7A	107.2 (11)	C13—C18—H18B	111.5 (12)
C6—C7—H7A	111.3 (11)	C13—C18—H18A	110.3 (17)
H7B—C7—H7A	105.5 (16)	H18B—C18—H18A	101 (2)
C4—C5—C6	122.20 (16)	C13—C18—H18C	114.8 (15)
C4—C5—H5	119.1 (11)	H18B—C18—H18C	102 (2)
C6—C5—H5	118.7 (11)	H18A—C18—H18C	116 (2)
C14—C13—C12	117.91 (15)	C11—C17—H17A	107 (3)
C14—C13—C18	122.03 (18)	C11—C17—H17C	111 (3)
C12—C13—C18	120.06 (17)	H17A—C17—H17C	91 (3)
N—C8—C9	112.46 (12)	C11—C17—H17B	112 (2)
N—C8—H8B	110.9 (11)	H17A—C17—H17B	104 (4)
C9—C8—H8B	108.7 (11)	H17C—C17—H17B	127 (4)
N—C8—H8A	105.3 (11)	C14—O2—H''	110.3 (18)
C9—C8—H8A	112.1 (10)		
			
O1—C1—C2—C3	179.02 (15)	C1—C6—C5—C4	1.4 (3)
C6—C1—C2—C3	0.6 (2)	C7—C6—C5—C4	−176.34 (16)
O1—C1—C2—C15	0.9 (2)	O2—C14—C13—C12	178.87 (15)
C6—C1—C2—C15	−177.59 (17)	C9—C14—C13—C12	0.8 (2)
O1—C1—C6—C5	−179.66 (14)	O2—C14—C13—C18	−0.5 (3)
C2—C1—C6—C5	−1.2 (2)	C9—C14—C13—C18	−178.61 (17)
O1—C1—C6—C7	−1.9 (2)	C19—N—C8—C9	−65.34 (18)
C2—C1—C6—C7	176.55 (15)	C7—N—C8—C9	170.58 (14)
C11—C10—C9—C14	0.9 (3)	C10—C9—C8—N	105.66 (17)
C11—C10—C9—C8	−177.98 (17)	C14—C9—C8—N	−73.2 (2)
O2—C14—C9—C10	−179.40 (15)	C9—C10—C11—C12	−0.2 (3)
C13—C14—C9—C10	−1.2 (2)	C9—C10—C11—C17	−179.2 (2)
O2—C14—C9—C8	−0.5 (2)	C6—C5—C4—C3	−0.9 (3)
C13—C14—C9—C8	177.70 (14)	C6—C5—C4—C16	177.5 (2)
C1—C2—C3—C4	0.0 (3)	C2—C3—C4—C5	0.2 (3)
C15—C2—C3—C4	178.09 (18)	C2—C3—C4—C16	−178.2 (2)
C19—N—C7—C6	67.71 (18)	C10—C11—C12—C13	−0.3 (3)
C8—N—C7—C6	−167.98 (14)	C17—C11—C12—C13	178.8 (2)
C5—C6—C7—N	−136.72 (16)	C14—C13—C12—C11	0.0 (3)
C1—C6—C7—N	45.6 (2)	C18—C13—C12—C11	179.35 (19)

### Hydrogen-bond geometry (Å, º)

**Table:**

*D*—H···*A*	*D*—H	H···*A*	*D*···*A*	*D*—H···*A*
O1—H′···N	1.03 (3)	1.71 (3)	2.6895 (18)	156 (2)
O2—H′′···O1^i^	0.94 (3)	2.02 (4)	2.9114 (18)	158 (2)

Symmetry code: (i) −*x*+1, −*y*+1, −*z*.
